# Phenotypic Clusters of Severe Asthma and Response to Dupilumab in Real‐World Practice

**DOI:** 10.1002/clt2.70198

**Published:** 2026-09-15

**Authors:** Daria S. Fomina, Sergey V. Fedosenko, Аnton А. Chernov, Elena N. Bobrikova, Оlga А. Mukhina, Marina S. Lebedkina, Alexander V. Karaulov, Asel Yu. Nurtazina, Mariana A. Lysenko, Harald Renz

**Affiliations:** ^1^ Moscow Clinical Research Center Hospital 52 of the Moscow Health Department Moscow Russia; ^2^ Federal State Autonomous Educational Institution of Higher Education “I.M. Sechenov First Moscow State Medical University of the Ministry of Health of the Russian Federation (Sechenov University)” Moscow Russia; ^3^ Federal State Budgetary Educational Institution “Siberian State Medical University” of the Ministry of Health of the Russian Federation Tomsk Russia; ^4^ Federal State Budgetary Educational Institution of Postgraduate Education “Russian Medical Academy of Continuous Professional Education” of the Ministry of Health of the Russian Federation Moscow Russia; ^5^ Federal State Budgetary Educational Institution of Higher Education “Pirogov Russian National Research Medical University” of the Ministry of Health of the Russian Federation Moscow Russia; ^6^ Institute of Laboratory Medicine and Pathobiochemistry Philipps University Marburg Marburg Germany; ^7^ University Hospital Giessen and Marburg Marburg Germany

**Keywords:** anti‐IL‐4/13R therapy, dupilumab, phenotypes, severe asthma, T2‐asthma

## Abstract

**Background:**

Severe asthma is a heterogeneous disease with variable clinical and inflammatory profiles that may influence responses to biological therapies. Identifying phenotypic clusters may help optimize patient selection and treatment outcomes.

**Objective:**

To identify phenotypic clusters (PCs) of patients with severe asthma treated with dupilumab and to assess differences in treatment response across clusters over a 12‐month period in a real‐world setting.

**Methods:**

This was a retrospective study involving 140 adult patients with severe, poorly controlled T2‐asthma who received dupilumab as first‐line biological therapy for at least 12 months. Agglomerative clustering was applied to baseline clinical, laboratory, and functional parameters to identify PCs. Treatment response was assessed at 1, 6, and 12 months using ACT, ACQ‐5, AQLQ, lung function (pre‐bronchodilator FEV_1_), T2‐biomarkers, and exacerbation rates.

**Results:**

Three phenotypic clusters were identified. PC1 (early‐onset allergic asthma with high IgE and severe atopic dermatitis) and PC3 (late‐onset mixed asthma with moderate IgE and persistent eosinophilia) showed rapid improvements in asthma control, lung function, and quality of life, with 71% and 75% of patients free from exacerbations, respectively. PC2 (predominantly non‐allergic, late‐onset, female‐dominant, with CRSwNP and NSAID hypersensitivity) demonstrated slower but progressive improvement, with 61.2% remaining exacerbation‐free after 12 months.

**Conclusion:**

This retrospective real‐world study identified distinct phenotypic clusters of severe asthma with differential responses to dupilumab. Early identification of these phenotypes may help guide personalized asthma management.

## 1 Introduction

Severe, poorly controlled asthma, though affecting only ∼3.7% of asthma patients, accounts for a disproportionate healthcare burden due to reduced quality of life, disability, and frequent severe exacerbations [1].

Currently approved biologics are primarily indicated for T2‐high allergic or non‐allergic phenotypes, except for tezepelumab, which has also shown efficacy in patients with low blood and airway eosinophils not receiving systemic corticosteroids (SCS) [2]. However, disease heterogeneity, driven by multiple cellular and cytokine pathways, can hinder optimal responses, even when therapies are selected according to guidelines [3].

Dupilumab, a fully human monoclonal antibody targeting the shared IL‐4/IL‐13 receptor *α*‐subunit, is indicated for moderate‐to‐severe T2‐asthma. Its clinical efficacy in improving asthma control, reducing exacerbations, and enhancing lung function and quality of life has been demonstrated in both randomized trials and real‐world studies [4, 5].

This retrospective real‐world study aimed to identify phenotypic clusters (PCs) among patients with severe asthma treated with dupilumab and to compare treatment responses across these groups over a 12‐month follow‐up.

## 2 Methods

### 2.1 Study Design and Participants

This was a retrospective comparative study including data of 140 adult patients (≥ 18 years), who had initiated dupilumab as first‐line biologic therapy for severe, poorly controlled asthma and had at least 12 months of follow‐up.

Data for the present study were retrieved from the “System for Personalized Dynamic Monitoring of Patients Receiving Biologic Therapies in Allergology and Immunology,” which is an ongoing prospective, single‐center observational registry established at Municipal Clinical Hospital No. 52 in Moscow in March 2021. The registry collects routine clinical practice data on adult patients with uncontrolled or partially controlled asthma and/or associated allergic and type 2 inflammatory diseases, including atopic dermatitis, allergic rhinitis, allergic conjunctivitis, chronic rhinosinusitis with nasal polyps (CRSwNP), and chronic urticaria, who initiate systemic biologic therapy. Patients are eligible for inclusion in the registry if they provide written informed consent and start treatment with a biologic agent used in routine allergology and clinical immunology practice, including omalizumab, mepolizumab, benralizumab, dupilumab, or tezepelumab. Eligibility for biologic treatment is determined in accordance with the applicable clinical guidelines of the Ministry of Health of the Russian Federation. The registry prospectively records demographic characteristics, disease history and phenotype, relevant comorbidities, previous and concomitant treatment, clinical measures of disease control, patient‐reported outcomes, pulmonary function, laboratory and type 2 inflammatory biomarkers, exacerbations, systemic corticosteroid exposure, biologic treatment, treatment effectiveness, and adverse events. Baseline data are collected at biologic treatment initiation, with subsequent clinical and laboratory assessments performed during routine follow‐up and entered into the registry by allergy specialists at prespecified assessment points relevant to the treated disease and biologic therapy.

For the present retrospective analysis, all consecutive patients from the registry who met the predefined eligibility criteria were included. All patients met criteria for poorly controlled disease (Asthma Control Test, ACT < 20; Asthma Control Questionnaire‐5, ACQ‐5 > 1.5) despite medium/high‐dose inhaled corticosteroids (ICS) in combination with a long‐acting β2‐agonist (LABA) ± long‐acting muscarinic antagonist (LAMA) or leukotriene receptor antagonist (LTRA). Inclusion also required ≥ 2 asthma exacerbations (treated with either a short course of SCS or high‐dose nebulized budesonide) during the previous year, evidence of T2‐inflammation (blood eosinophils ≥ 150/μL, sputum eosinophils ≥ 2%, FeNO ≥ 20 ppb, or clinically relevant allergic sensitization), or regular use of SCS (> 50% of the year) [6]. Allergic sensitization was assessed using skin testing and serum allergen‐specific IgE measurement (ImmunoCAP, Thermo Fisher Scientific) and was considered clinically relevant when test results were consistent with the patient's clinical history and allergen exposure.

### 2.2 Ethics

The study was approved by the Ethics Committee of Municipal Clinical Hospital No. 52 (protocol No. 01вн/0221, March 3, 2021) and conducted in accordance with the Declaration of Helsinki. All patients provided written informed consent for the collection and research use of their de‐identified data as part of the registry protocol. As this was a retrospective analysis of pre‐existing de‐identified data from a scientific registry, no additional consent was required.

### 2.3 Treatment and Follow‐Up

Dupilumab was administered subcutaneously (600 mg loading dose followed by 300 mg every 2 weeks). Treatment response was assessed at baseline (V0), 1 month (V1), 6 months (V2), and 12 months (V3).

### 2.4 Variables and Preliminary Analysis

At the first step of the study, descriptive statistical analysis of both qualitative and quantitative variables was conducted using the IBM SPSS Statistics V‐23.

Candidate baseline variables with no more than 30% missing data were considered for clustering. This threshold was selected a priori as a pragmatic compromise between preserving clinically relevant information and minimizing the potential impact of extensive missing data on the clustering procedure.

Associations between variables were then assessed as an initial feature selection step using statistical methods appropriate for the respective variable characteristics. Variables demonstrating at least a weak‐to‐moderate association (|*r*| ≥ 0.3, *p* < 0.05) were retained for clustering. This correlation threshold was selected a priori to preserve informative variables while excluding variables with negligible relationships that were unlikely to contribute meaningfully to cluster formation.

Multicollinearity among the retained variables was subsequently assessed using a pairwise Pearson correlation matrix. Pairs of independent variables with an absolute Pearson correlation coefficient (|*r*| > 0.7) were considered potentially collinear and were reviewed to minimize redundancy before model development. The following list of baseline parameters was incorporated into the clustering analysis (Supporting Information S1: Table S1): demographics, features of asthma history, smoking history, baseline results of ACQ‐5, ACТ, and AQLQ (Asthma Quality of Life Questionnaire), T2‐biomarkers (absolute blood eosinophil count (BEC), total serum immunoglobulin E (tIgE), FeNO), pre‐bronchodilator forced expired volume for the first second (pre‐FEV_1_) and post‐bronchodilator FEV_1_ to FVC (forced vital capacity; post‐ FEV_1_/FVC) ratio, evidence of the “Atopic March” (i.e., progression of allergic diseases that often begin in early childhood, moving from eczema, to food allergies, and later to asthma and allergic rhinitis), severe atopic dermatitis (SAD), CRSwNP, allergic sensitization patterns, history of allergen‐specific immunotherapy (ASIT; recorded as any previous documented course of treatment, irrespective of duration or the time elapsed since its completion), non‐steroid anti‐inflammatory drugs (NSAIDs) hypersensitivity, comorbidities, and conventional asthma phenotypes classified according to the diagnostic categories of the International Statistical Classification of Diseases and Related Health Problems, 10th Revision (ICD‐10) [7]—allergic (J45.0), non‐allergic (J45.1), and mixed asthma (J45.8). The following baseline variables were excluded from the analysis: history of polypectomy, food allergy type (specific food allergen), basophil count, and baseline Sino‐Nasal Outcome Test‐22 (SNOT‐22) score.

### 2.5 Data Preprocessing and Clustering Analysis

Phenotypic clusters (PCs) were identified using agglomerative hierarchical clustering [8, 9, 10]. Quantitative variables were scaled prior to clustering: normally distributed variables were standardized using StandardScaler, whereas non‐normally distributed variables were scaled using MinMaxScaler [11]. Missing values were handled using simple imputation: categorical variables were imputed with the mode, and numerical variables with the mean (for normally distributed variables) or median (for non‐normally distributed variables).

The optimal number of clusters was determined using internal validation metrics (Davies–Bouldin, Calinski–Harabasz, and silhouette indices) [12, 13, 14], evaluated across candidate solutions ranging from 2 to 9 clusters. The final 3‐cluster solution was selected based on the overall balance of these metrics (Supporting Information S1: Table S2).

For dimensionality reduction and visualization only, t‐distributed stochastic neighbor embedding (t‐SNE) was applied to the same preprocessed baseline dataset [15]. The t‐SNE projection was not used for cluster assignment; instead, cluster labels obtained from agglomerative clustering in the original feature space were overlaid onto the two‐dimensional t‐SNE map. Perplexity (reflecting the effective number of nearest neighbors) was optimized by minimizing Kullback–Leibler (KL) divergence across candidate values from 5 to 50 [16]. The selected perplexity value was 15, corresponding to a KL divergence of 0.66.

### 2.6 Final Comparative Analysis and Treatment Outcomes

The identified PCs were subsequently subjected to comparative analysis of anti–IL‐4/13R treatment outcomes. Treatment response was assessed at baseline (V0) and after 1 (V1), 6 (V2), and 12 (V3) months of therapy based on changes across the following domains:

- *Asthma control level*: proportions of patients with ACQ‐5 ≥ 1.5 (*uncontrolled*), < 1.5 (*partially and well controlled*) and < 0.75 (*well controlled*); proportions of patients achieving ACТ scores of < 20 (*uncontrolled*), ≥ 20 (*partially and well controlled*) and 25 (*well controlled*);
- *Pulmonary function*: proportions of patients with pre‐bronchodilator FEV_1_ < 65% (*significant reduction*), ≥ 65% (*normal or moderately reduced*) and ≥ 80% (*optimal*) of predicted;
- *Quality of life*: proportions of patients with AQLQ increase by ≥ 0.5 of baseline and/or AQLQ of 7;
- *Reduction of asthma exacerbation risk* (proportions of patients without asthma exacerbations that required steroid treatment escalation, including the administration of SCS or high‐dose nebulized budesonide in addition to regular maintenance therapy).

Continuous variables were presented as median (P25‐P75). Categorical variables were presented as frequencies and percentages. Group comparisons were performed using the Mann–Whitney *U* test for continuous variables and the χ^2^ or Fisher's exact test for categorical variables, as appropriate. Post‐hoc pairwise comparisons were performed using Dunn's test with Holm correction for multiple testing. A *p*‐value < 0.05 was considered statistically significant.

## 3 Results

The study included 140 patients (48.6% female), with a median age of 43.0 (32.0–58.3) years and asthma duration of 18 (10–28.3) years prior to biologic therapy initiation. Smoking index among current and former smokers was 7.0 (2.0–15.5) pack‐years. Obesity was present in 29 patients (20.7%).

Baseline asthma control was poor (ACQ‐5 2.8 (1.6–3.8); ACT 15.0 (11.0–19.0)), with moderate impairment in quality of life (AQLQ 4.2 ± 1.4). Baseline pre‐FEV_1_ was 81.0 (66.0–94.0)% predicted, and post‐FEV1/FVC ratio was 0.76 (0.68–0.84).

Baseline T2‐biomarkers showed a wide distribution, with levels of blood eosinophils 500.0 (295.0–800.0)/μL, total IgE 322.0 (95.2–2000.0) IU/mL, and FeNO 24.0 (14.0–44.0) ppb.

### 3.1 Identification of PCs and Baseline Comparative Analysis

**FIGURE 1:**
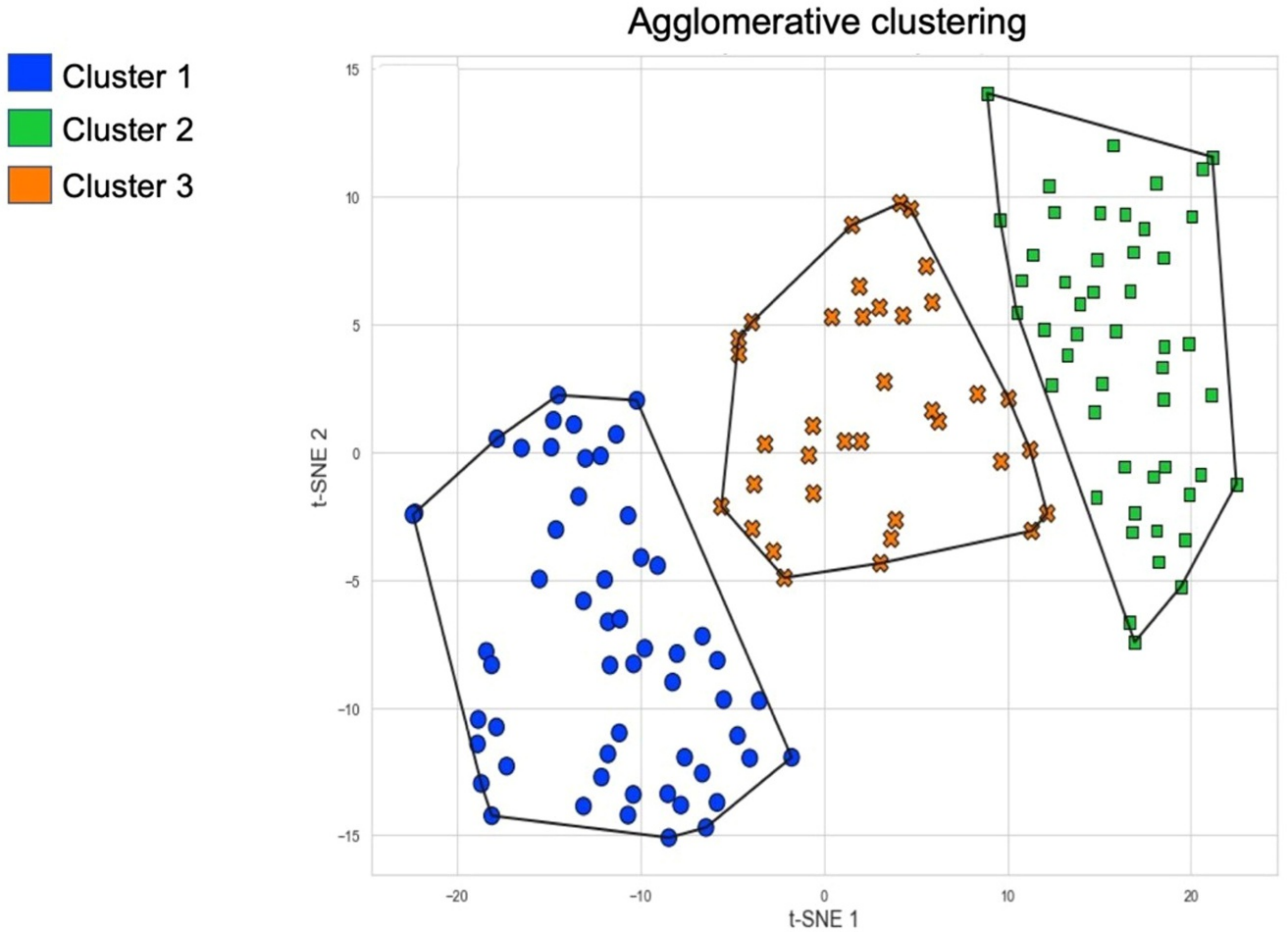
Visualization of severe asthma phenotypic clusters among patients treated with dupilumab, using t‐SNE dimensionality reduction. T‐SNE, t‐distributed Stochastic Neighbor Embedding; tSNE 1 and 2 are hyperparameters. Two‐dimensional t‐SNE visualization of three phenotypic clusters identified by agglomerative hierarchical clustering in the original multidimensional baseline feature space. Each point represents one patient, with colors indicating cluster assignment. Clustering was based on clinical, functional, and T2‐inflammatory biomarker parameters.

Agglomerative clustering identified three distinct PCs, as visualized by t‐SNE (Figure 1). Baseline characteristics are summarized in Table 1.

**TABLE 1:** Analysis of quantitative parameters depending on severe asthma phenotypes.

Parameters Me (P25‐P75)	PC1 55 (39.3%)	PC2 49 (35.0%)	PC3 36 (25.7%)	p*	*P*_PC1‐PC2_	*P*_PC1‐PC3_	*P*_PC2‐PC3_
Age at biological treatment initiation, years	32 (22; 42)	56 (44; 66)	45 (36; 61)	< 0.0001	< 0.0001	< 0.0001	0.026
Age at asthma onset, years	6 (4; 14)	36 (29; 45)	29 (13; 40)	< 0.0001	< 0.0001	< 0.0001	< 0.0001
Smoking index, pack‐years	6 (1; 15), *n* = 13	5 (2; 10), *n* = 14	16 (9; 25), *n* = 8	0.133	NA	NA	NA
Patients on SCS > 50% of 12 months prior to biological treatment initiation, *n* (% of all patients)	9 (16.4%)	17 (34.7%)	4 (11.1%)	0.016	0.031	> 0.05	0.013
ACQ5 score	2.7 (1.2; 3.8), *n* = 54	3 (2; 4.1), *n* = 47	2 (1.3; 3), *n* = 35	0.012	> 0.05	> 0.05	0.01
ACT score	15 (11; 19)	13 (9; 16)	16 (12; 19), *n* = 35	0.017	0.036	> 0.05	0.036
SNOT‐22 score	36 (13; 58), *n* = 6	57 (51; 63), *n* = 43	56 (47; 64), *n* = 29	0.063	NA	NA	NA
AQLQ score	4.56 (4.16; 4.97), *n* = 54	3.66 (3.28; 4.03), *n* = 47	4.34 (4; 4.72), *n* = 35	0.004	0.005	> 0.05	0.027
Eosinophils, cells/μL	625 (410; 1078), *n* = 54	400 (240; 600), *n* = 48	590 (312; 893)	0.005	0.004	> 0.05	> 0.05
Total IgE, IU/mL	2000 (726; 3166), *n* = 54	93 (35; 256), *n* = 48	200 (96; 690), *n* = 35	< 0.0001	< 0.0001	< 0.0001	> 0.05
FeNO, ppb	22 (12; 38), *n* = 44	24 (11; 45), *n* = 44	30 (20; 53), *n* = 29	0.223	NA	NA	NA
Pre‐bronchodilator FEV_1_, % of predicted	82 (77; 88), *n* = 53	75 (70; 80), *n* = 49	82 (74; 89), *n* = 35	0.139	NA	NA	NA
Post‐bronchodilator FEV_1_/FVC ratio	78 (74; 82), *n* = 52	74 (70; 78), *n* = 48	74 (69; 80), *n* = 34	0.364	NA	NA	NA

*Note:* Overall p* values were obtained using the Kruskal–Wallis test for quantitative variables and the χ^2^ test for categorical variables. Pairwise comparisons were performed only for variables with a significant overall test. Non‐significant pairwise comparisons are reported as *p* > 0.05.

Abbreviations: μL, microliter; ACQ‐5, Asthma Control Questionnaire‐5; ACT, Asthma Control Test; AQLQ, Asthma Quality of Life Questionnaire; FeNO, fractional exhaled nitric oxide; FEV_1_, forced expiratory volume in 1 second; FVC, forced vital capacity; IgE, immunoglobulin E; IU/mL, international units per milliliter; Me, median; NA, not applicable; P25–P75, 25th and 75th percentiles; PC1, PC2, PC3, phenotypic clusters 1, 2, and 3; ppb, parts per billion; SCS, systemic corticosteroids; SNOT‐22, Sinonasal Outcome Test‐22.

Clusters differed significantly in age at asthma onset and treatment initiation. PC1 included predominantly younger patients (80% < 45 years; 3.6% > 59 years), whereas PC2 had the highest proportion of older patients (30.6% aged 45%–59% and 42.9% ≥ 60 years), and PC3 showed an intermediate distribution (22.2% aged 45%–59% and 30.6% ≥ 60 years); *p* < 0.001.

Sex distribution also differed (*p* < 0.0001): PC1 was predominantly male (72.7%, *n* = 40), PC2 predominantly female (71.4%, *n* = 35), while PC3 showed equal male and female representation.

Asthma phenotypes (International Classification of Diseases, 10/11 Revision) varied markedly across clusters. Allergic asthma predominated in PC1 (85.5%, *n* = 47) compared with PC2 (10.2%, *n* = 5) and PC3 (41.7%, *n* = 15); *p* < 0.001. Non‐allergic asthma was most frequent in PC2 (87.8%, *n* = 43) versus PC1 (0%) and PC3 (5.6%, *n* = 2); *p* < 0.001. PC3 had the highest proportion of mixed asthma (52.7%, *n* = 19), exceeding PC1 (14.5%, *n* = 8) and PC2 (2.0%, *n* = 1); *p* < 0.001. Differences were significant between all groups except PC1 versus PC3 for non‐allergic asthma (*p* > 0.05).

At baseline (V0), no patients had well‐controlled asthma (ACT = 25 and/or ACQ‐5 < 0.75). PC2 demonstrated poorer asthma control, with higher ACQ‐5 and lower ACT scores, and lower AQLQ scores than PC1 and PC3 (Table 1). All clusters showed median BEC consistent with marked eosinophilia (≥ 400 cells/μL). However, low BEC (< 150 cells/μL) was observed in a minority of patients: 9.4% in PC1 and 14.3% in PC2. Peripheral eosinophilia ≥ 300 cells/μL prior to dupilumab initiation was present in 83.1% of PC1, 65.3% of PC2, and 75.0% of PC3 patients (*p* = 0.065). PC1 was characterized by higher BEC compared with PC2 and a pronounced high‐IgE profile, with median total IgE levels approximately 10‐fold higher than in PC3 and 21.5‐fold higher than in PC2. All patients in PC1 had tIgE > 30 IU/mL, whereas levels < 30 IU/mL were observed in 20.8% of PC2 and 5.7% of PC3 (*p* = 0.001). FeNO values did not differ significantly between clusters (Table 1).

Baseline pulmonary function was comparable across groups (Table 1). Pre‐FEV_1_ ≥ 65% predicted was observed in 84.9% of PC1, 71.4% of PC2, and 71.4% of PC3 patients (*p* = 0.192).

**FIGURE 2:**
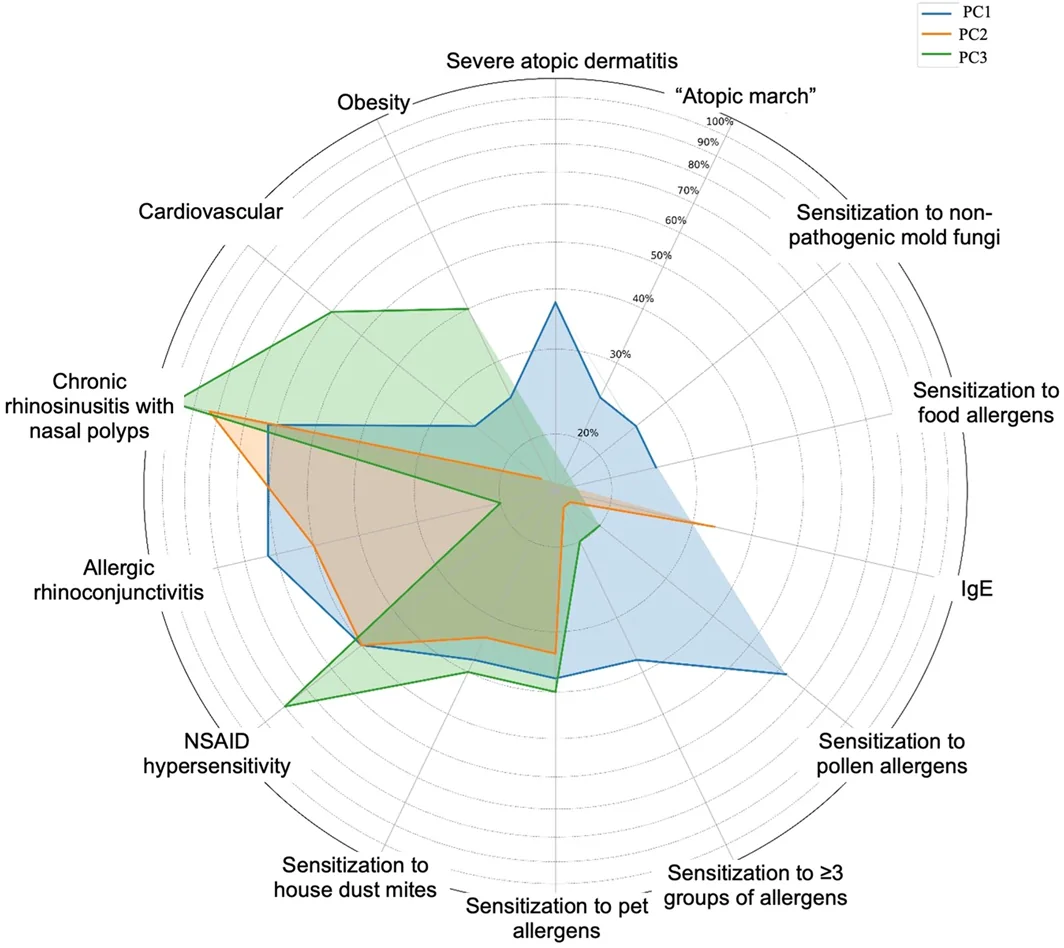
Radar analysis comparing phenotypic characteristics of patients with severe asthma treated with dupilumab. The analysis highlights differences in allergic sensitization patterns, comorbid conditions, and immunological profiles. PC1 is characterized by severe atopic dermatitis, high total IgE, and multiple allergen sensitizations consistent with the “Atopic march” concept (a progressive pattern of allergic disease development, typically beginning with atopic dermatitis in infancy, followed by allergic rhinitis and asthma later in life). PC2 represents predominantly non‐allergic asthma with lower IgE levels and frequent chronic rhinosinusitis with nasal polyps and NSAID hypersensitivity. PC3 demonstrates a mixed allergic profile with moderate IgE and persistent eosinophilia. These phenotypic differences may contribute to variations in response to anti‐IL4/13R therapy. IgE, immunoglobulin E; NSAID, non‐steroidal anti‐inflammatory drugs; PC1‐3, Phenotypic clusters 1–3.

The higher IgE profile of PC1 was further supported by a high prevalence of atopic comorbidities, distinguishing this phenotype from the others (Table 2; Figure 2).

**TABLE 2:** Confirmed AS, NSAID hypersensitivity, comorbidities or concurrent conditions.

Characteristics, *n* (% of cohort)	PC1, *n* = 55	PC2, *n* = 49	PC3, *n* = 36	*P* **	*P*_PC1‐PC2_	*P*_PC1‐PC3_	*P*_PC2‐PC3_
AS to house dust mites	52 (94.5%)	7 (14.3%)	18 (50.0%)	< 0.001	< 0.001	< 0.001	< 0.001
AS to pet allergens	53 (96.4%)	4 (8.2%)	21 (58.3%)	< 0.001	< 0.001	< 0.001	< 0.001
AS to pollen	52 (94.5%)	1 (2.0%)	23 (63.9%)	< 0.001	< 0.001	< 0.001	< 0.001
AS to allergens of non‐pathogenic mold fungi	32 (58.2%)	2 (4.1%)	2 (5.6%)	< 0.001	< 0.001	< 0.001	0.751
AS to food allergens	38 (69.1%)	0 (0.0%)	3 (8.3%)	< 0.001	< 0.001	< 0.001	0.040
AS to ≥ 3 groups of allergens^a^	55 (100.0%)	1 (2.0%)	9 (25.0%)	< 0.001	< 0.001	< 0.001	0.001
History of ASIT	6 (10.9%)	0 (0.0%)	2 (5.6%)	0.057	NA	NA	NA
AD as the initial clinical manifestation of AS	38 (69.1%)	0 (0.0%)	0 (0.0%)	< 0.001	< 0.001	< 0.001	1.000
ARC	55 (100.0%)	6 (12.2%)	30 (83.3%)	< 0.001	< 0.001	0.002	< 0.001
Severe AD	55 (100.0%)	1 (2.0%)	6 (16.7%)	< 0.001	< 0.001	< 0.001	0.015
NSAID hypersensitivity	1 (1.8%)	28 (57.1%)	17 (47.2%)	< 0.001	< 0.001	< 0.001	0.365
CRSwNP	6 (10.9%)	43 (87.8%)	29 (80.6%)	< 0.001	< 0.001	< 0.001	0.362
Chronic urticaria	2 (3.6%)	2 (4.1%)	1 (2.8%)	0.950	NA	NA	NA
GERD	9 (16.4%)	7 (14.3%)	1 (2.8%)	0.129	NA	NA	NA
Cardiovascular diseases	6 (10.9%)	18 (36.7%)	9 (25.0%)	0.008	0.002	0.076	0.251
Diabetes mellitus	0 (0.0%)	3 (6.1%)	2 (5.6%)	0.185	NA	NA	NA
Obesity	3 (5.5)	15 (30.6)	11 (30.6)	0.002	< 0.001	0.001	0.996

Abbreviations: AD, atopic dermatitis; ARC, allergic rhinoconjunctivitis; AS, allergic sensitization; ASIT, allergen‐specific immunotherapy; CRSwNP, chronic rhinosinusitis with nasal polyps; GERD, gastroesophageal reflux disease; NA, not applicable; NSAID, non‐steroidal anti‐inflammatory drugs; PC1, PC2, PC3, phenotypic clusters 1, 2, and 3.

^a^ AS to ≥ 3 groups of allergens was defined as sensitization to ≥ 3 of the following allergen groups: house dust mites, pet allergens, pollen, non‐pathogenic mold fungi, and food allergens.

^**^ Overall *p* values were calculated using the *χ* ^2^ test. Pairwise comparisons were performed only for variables with a significant overall test. Non‐significant pairwise comparisons are reported as *p* > 0.05.

Notably, patients in PC2 more frequently required systemic corticosteroids for > 50% of the year prior to biologic initiation compared with PC1 and PC3 (Table 1).

### 3.2 Anti‐IL‐4/13R Treatment Outcomes Across Clusters

Dupilumab treatment was associated with a marked reduction in asthma exacerbations across all clusters. During 12 months of follow‐up, no exacerbations were recorded in 70.9% (PC1), 61.2% (PC2), and 75.0% (PC3) of patients (*p* = 0.36).

**FIGURE 3:**
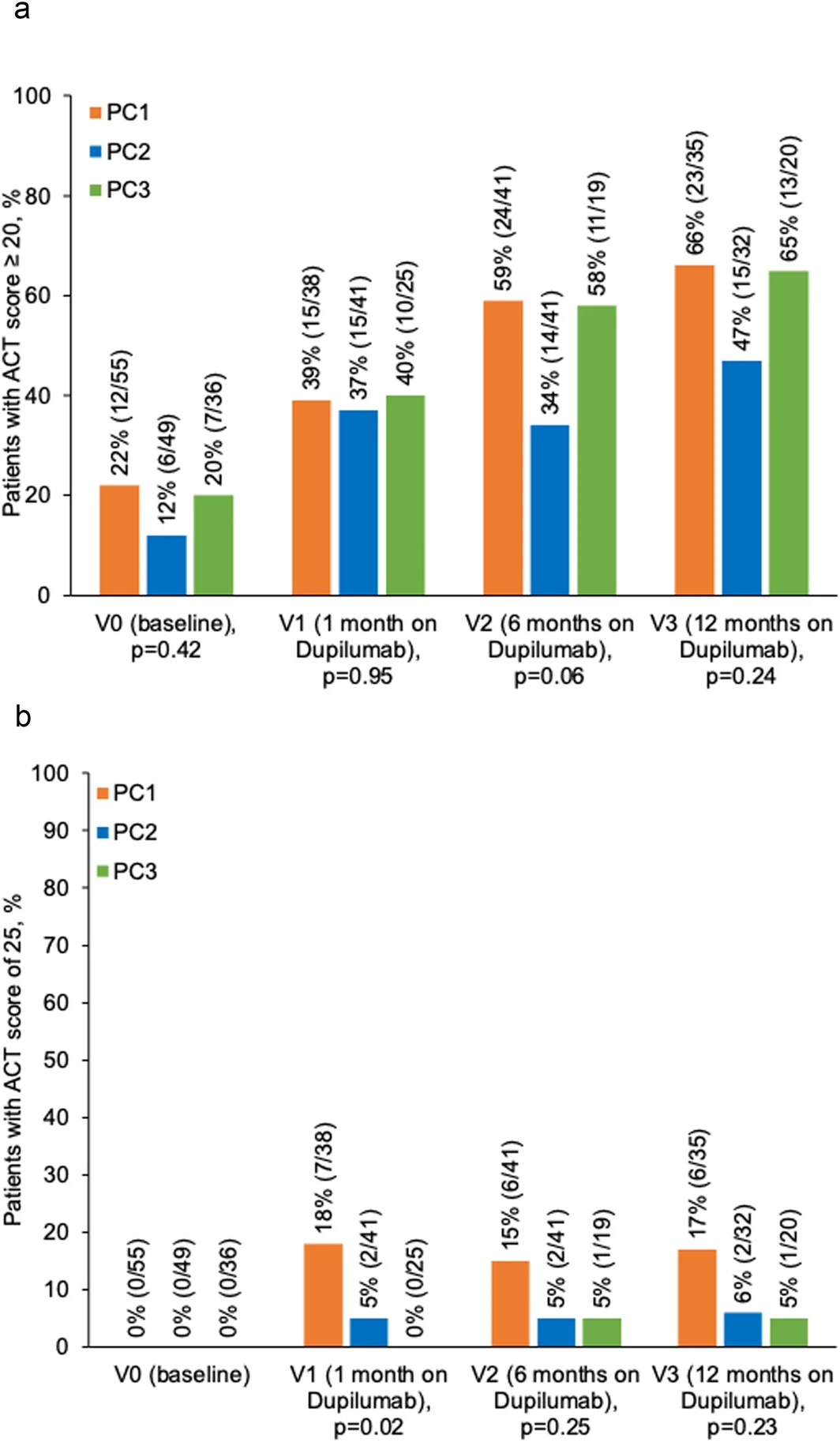
The proportion of patients achieving asthma control according to ACT scores during dupilumab therapy. (a) The percentage of patients with an Asthma Control Test (ACT) score ≥ 20, indicating partial or complete asthma control, at baseline (V0), 1 month (V1), 6 months (V2), and 12 months (V3). (b) The percentage of patients achieving an ACT score of 25, representing full asthma control, at the same time points. Data are shown for phenotypic clusters PC1, PC2, and PC3. PC1 and PC3 demonstrate a more rapid improvement in asthma control, whereas PC2 exhibits a more gradual response over the 12‐month period. ACT, Asthma Control Test; PC, phenotypic cluster; V0, baseline; V1, 1 month; V2, 6 months; V3, 12 months.

**FIGURE 4:**
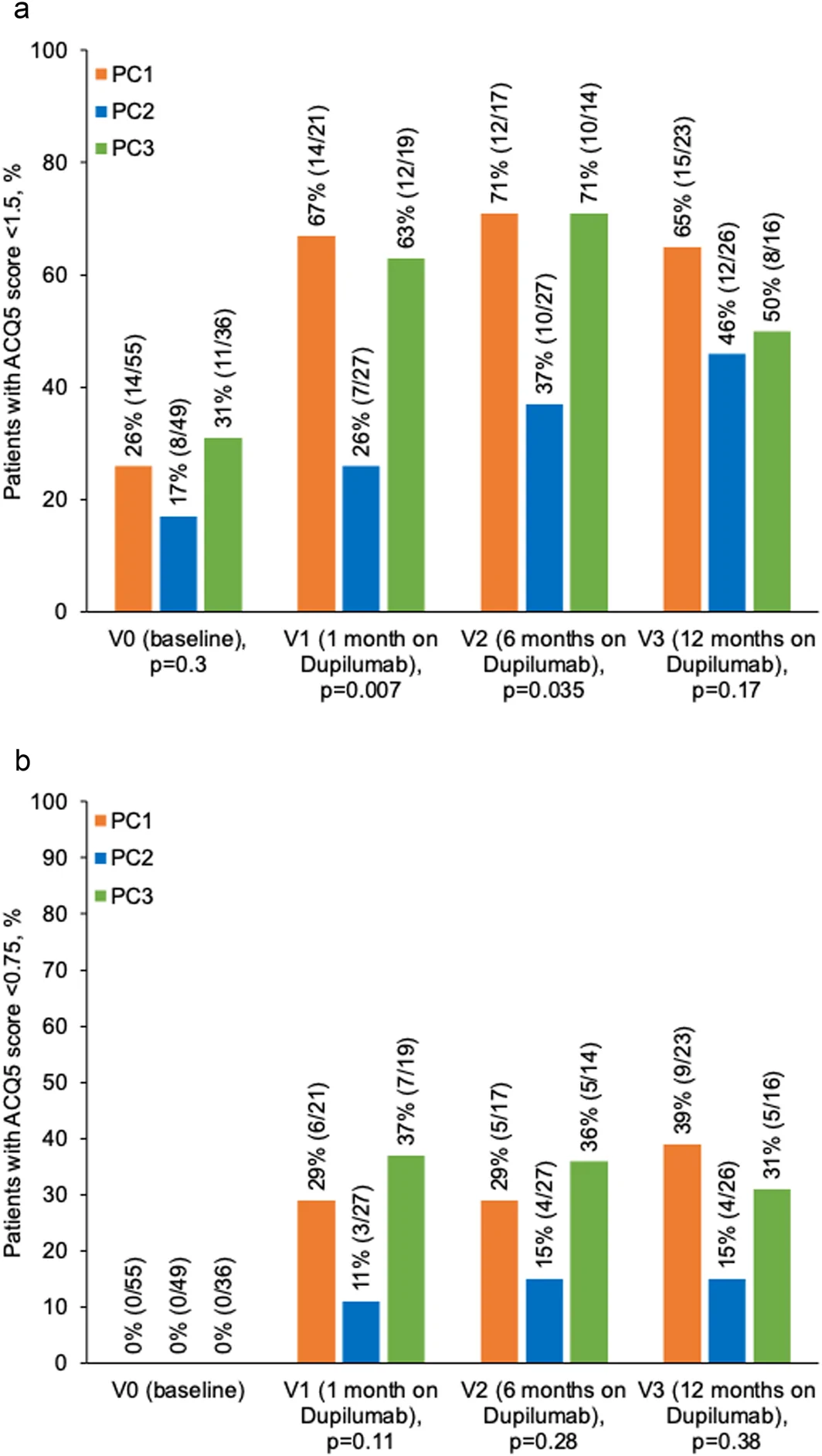
The proportion of patients achieving asthma control according to ACQ5 scores during dupilumab therapy. (a) The percentage of patients with an Asthma Control Questionnaire‐5 (ACQ5) score < 1.5, indicating partial or complete asthma control, at baseline (V0), 1 month (V1), 6 months (V2), and 12 months (V3). (b) The percentage of patients achieving an ACQ5 score < 0.75, reflecting well‐controlled asthma, at the same time points. PC1 and PC3 show earlier improvements in asthma control, while PC2 demonstrates a more gradual response over 12 months of therapy. ACQ5, Asthma Control Questionnaire‐5; PC, phenotypic cluster; V0, baseline; V1, 1 month; V2, 6 months; V3, 12 months.

Asthma control, as measured by ACT and ACQ‐5, improved in all clusters. PC1 and PC3 demonstrated more rapid responses, while PC2 showed a slower but progressive improvement (Figures 3 and 4). The highest rates of complete asthma control (ACT = 25) were observed in PC1 at 1, 6, and 12 months (18%, 15%, and 17%, respectively; Figure 3b). At 12 months, ACQ‐5 < 1.5 was observed in 65% of PC1 and 50% of PC3 patients (Figure 4a). For well‐controlled asthma (ACQ‐5 < 0.75), rates at V3 were 39% (PC1), 15% (PC2), and 31% (PC3) (*p* = 0.38; Figure 4b).

**FIGURE 5:**
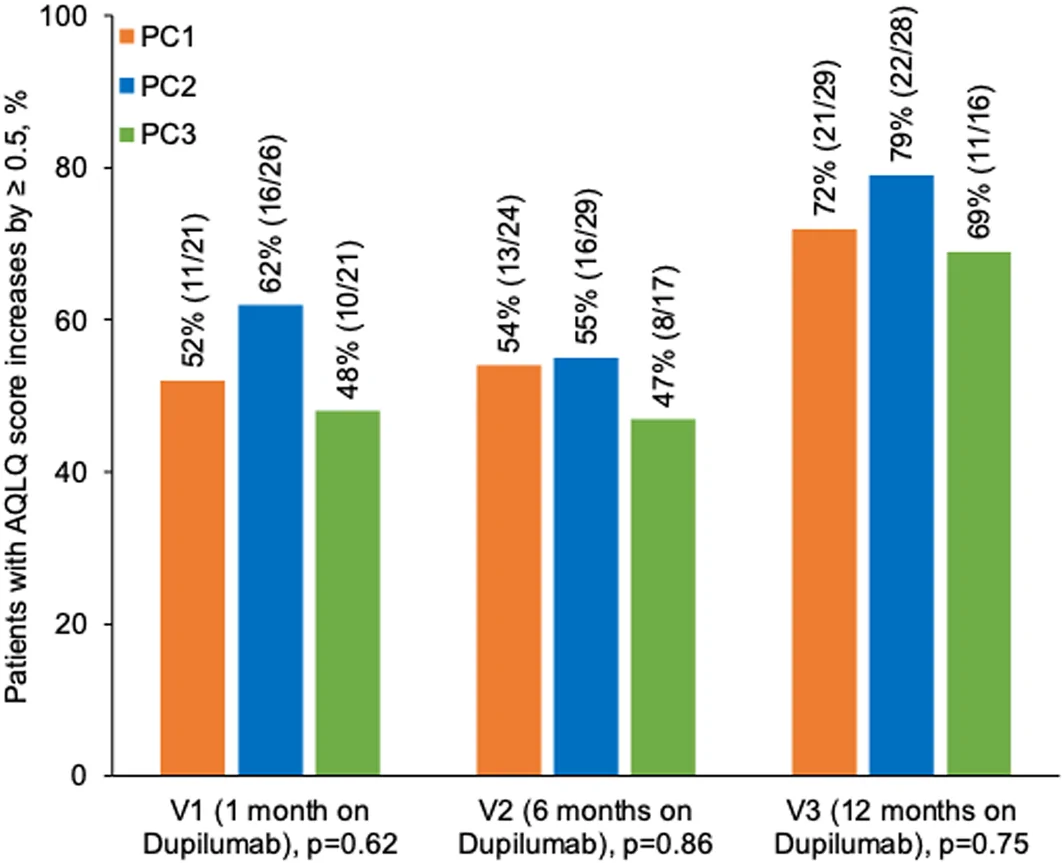
The proportion of patients experiencing an improvement in quality of life as measured by the Asthma Quality of Life Questionnaire (AQLQ) during dupilumab therapy. The percentage of patients with an AQLQ score increase of ≥ 0.5, reflecting a clinically meaningful improvement, is shown for phenotypic clusters (PC) 1, 2, and 3 at 1 month (V1), 6 months (V2), and 12 months (V3) after therapy initiation. PC1 and PC3 demonstrate earlier and sustained quality‐of‐life improvements, whereas PC2 shows a more gradual response over the treatment period. AQLQ, Asthma Quality of Life Questionnaire; PC, phenotypic cluster; V1, 1 month; V2, 6 months; V3, 12 months.

Quality of life improved across all clusters, with clinically meaningful AQLQ improvement (≥ 0.5) observed already at V1 in 52% (PC1), 62% (PC2), and 48% (PC3) of patients (*p* = 0.62), and in the majority of patients by 12 months without significant intergroup differences (Figure 5).

**FIGURE 6:**
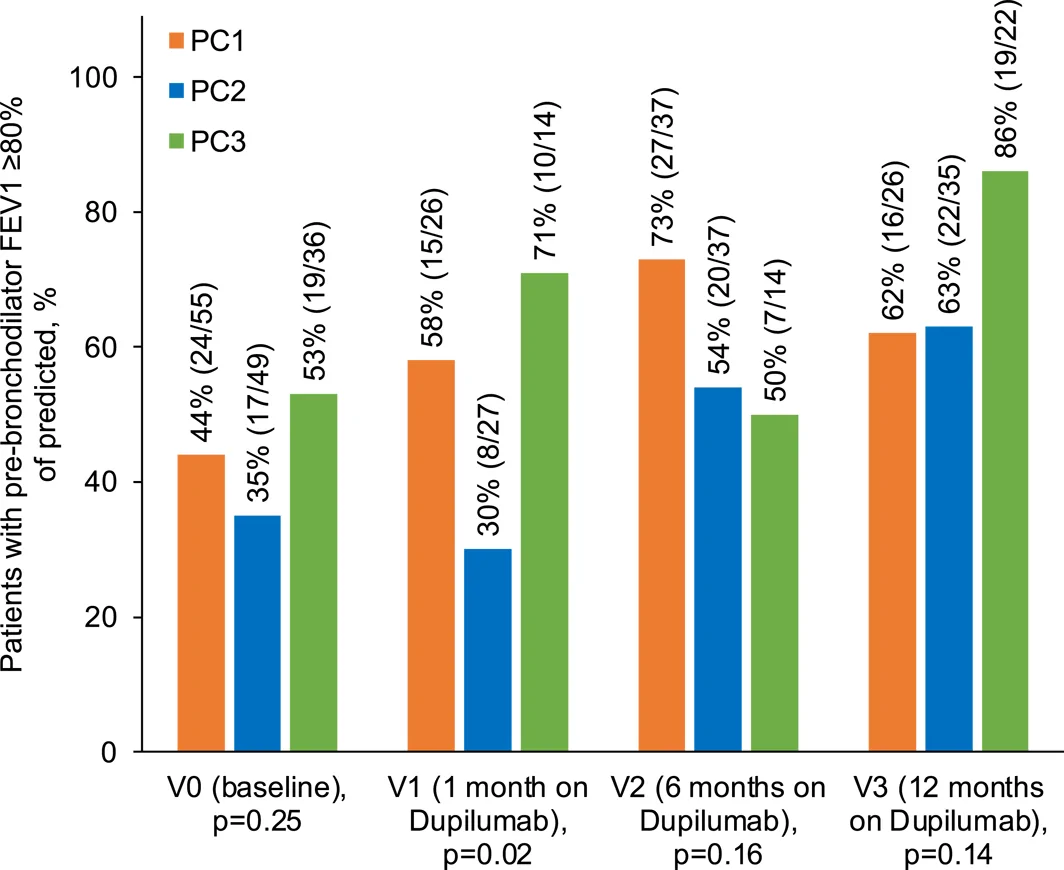
Pulmonary function assessment throughout the course of dupilumab therapy. The percentage of patients with a pre‐bronchodilator forced expiratory volume in one second (FEV_1_) ≥ 80% of predicted is shown for phenotypic clusters (PC) 1, 2, and 3 at baseline (V0), 1 month (V1), 6 months (V2), and 12 months (V3) after therapy initiation. PC1 and PC3 demonstrate a more rapid improvement in lung function, whereas PC2 exhibits a delayed but progressive increase in the proportion of patients reaching the FEV_1_ threshold. FEV_1_, forced expiratory volume in one second; PC, phenotypic cluster; V0, baseline; V1, 1 month; V2, 6 months; V3, 12 months.

Lung function improved in all clusters, with differing response kinetics (Figure 6). PC1 and PC3 showed earlier improvement in pre‐FEV_1_, whereas PC2 demonstrated a delayed but gradual increase. Early differences were observed across clusters at 1 month but were no longer significant at later time points. By 12 months, pre‐FEV_1_ ≥ 65% predicted was observed in 81% (PC1), 86% (PC2), and 91% (PC3), and pre‐FEV_1_ ≥ 80% predicted was observed in 62%, 63%, and 86% of patients, respectively (Figure 6).

## 4 Discussion

In this real‐world study, unsupervised clustering identified three clinically distinct phenotypic groups with different baseline characteristics and response patterns over 12 months. While all clusters demonstrated substantial clinical benefit, including a reduction in exacerbation burden, they differed in response trajectories across clinical domains. Specifically, patients in PC1 and PC3 showed earlier improvements in asthma control and lung function, whereas PC2 exhibited a slower but progressive response, despite comparable outcomes at later time points.

These findings are consistent with previous clustering studies in severe asthma, which have demonstrated substantial clinical and biological heterogeneity across phenotypes [17, 18], as well as with studies showing that dupilumab improves exacerbation outcomes, lung function, asthma control, and patient‐reported outcomes in type 2 asthma, and that baseline inflammatory and clinical characteristics influence the magnitude of response [4, 5, 19, 20, 21, 22, 23, 24, 25]. Our data further extend these observations by demonstrating that different phenotypic clusters may exhibit distinct response trajectories over time.

PC1 and PC3 may be interpreted as the two more rapidly responding phenotypes in our cohort despite differing clinical profiles. PC1 comprised an early‐onset, predominantly male phenotype with mainly allergic rather than mixed asthma, extensive polysensitization to perennial allergens, a high burden of atopic comorbidities (particularly allergic rhinitis and frequent severe atopic dermatitis) and minimal CRSwNP and NSAID hypersensitivity. In contrast, PC3 showed a more balanced sex distribution and a mixed/allergic profile with moderate IgE production, persistent eosinophilia, frequent allergic rhinoconjunctivitis and CRSwNP, a lower prevalence of severe atopic dermatitis, and intermediate patterns of sensitization. Despite these phenotypic differences, both clusters demonstrated similarly early improvement in asthma control and lung function, consistent with clinically relevant T2 inflammatory activity. PC1 aligns with a more overtly allergic, high‐IgE phenotype, whereas PC3 reflects a mixed eosinophilic–upper‐airway phenotype; however, both remain compatible with a favorable early response to IL‐4/IL‐13 receptor blockade. While PC1 included high IgE levels and extensive allergic sensitization, current evidence suggests that these features alone are not reliable predictors of response to dupilumab, as treatment efficacy has been demonstrated irrespective of allergic status [4, 26]. This is consistent with evidence that dupilumab provides greater clinical benefit in patients with more evident type 2 inflammatory features, particularly higher baseline FeNO and blood eosinophils, while retaining efficacy across a broad spectrum of type 2 phenotypes, including those with upper‐airway comorbidity such as CRSwNP [19, 24, 27, 28].

PC2 showed a delayed but progressive improvement in asthma control, quality of life, and lung function over 12 months, while still achieving clinically meaningful benefit and substantial exacerbation reduction by the end of follow‐up. This phenotype was characterized by late‐onset, largely non‐allergic asthma, female predominance, poorer baseline control and quality of life, frequent CRSwNP and NSAID hypersensitivity, lower eosinophil levels than PC1, and the highest baseline use of SCS. This profile may help explain the delayed response trajectory. Long‐term corticosteroid dependence in severe asthma is associated with greater disease burden and a more treatment‐refractory clinical course [29, 30, 31, 32, 33]. In addition, in long‐standing severe asthma, airway remodeling and fixed airflow limitation may limit the speed of functional improvement despite effective anti‐inflammatory therapy [33, 34, 35]. At the same time, the combination of CRSwNP and NSAID hypersensitivity in PC2 is compatible with a non‐allergic, yet T2–associated, airway disease pattern in which IL‐4/IL‐13 blockade remains clinically effective [4, 20]. These findings may also have practical implications for treatment evaluation. Patients with a delayed‐response phenotype, such as PC2, may require longer assessment before treatment efficacy can be reliably judged, thereby avoiding premature discontinuation or switching of biologic therapy.

Notably, all clusters showed a marked reduction in exacerbation burden, with no significant differences in the proportion of patients remaining exacerbation‐free at 12 months despite distinct baseline phenotypes and differences in response kinetics across clinical domains. This is consistent with evidence that dupilumab reduces exacerbations across a broad spectrum of T2 asthma phenotypes, including patients with varying baseline inflammatory and clinical characteristics [4, 19, 20].

This study has several limitations. Its retrospective design may introduce bias despite efforts to standardize patient selection and data processing. Conducted in a single specialized center, the findings may have limited generalizability to broader asthma populations with different healthcare systems, environmental exposures, or genetic backgrounds. The clustering was based on a predefined set of clinical, functional, and biomarker variables, which may not fully capture the heterogeneity of asthma. In addition, missing data may have influenced clustering results, as distance‐based methods are sensitive to incomplete observations and feature scaling; although only variables with ≤ 30% missingness were included and simple imputation (mean/median for numerical variables and mode for categorical variables) was applied, its potential impact on cluster structure and stability cannot be fully excluded. Finally, treatment adherence and variations in concomitant therapies, including inhaled corticosteroids, were not systematically assessed and may have influenced outcomes. In routine clinical practice within the registry, adherence was monitored indirectly through scheduled follow‐up visits, prescription records, and physician assessment; however, no standardized or objective adherence measures were consistently applied. Future prospective studies with larger, more diverse populations and longer follow‐up are needed to validate these findings and refine phenotypic classifications.

## 5 Conclusion

In this retrospective study, three distinct PCs of severe asthma were identified, all demonstrating substantial benefit from dupilumab, including a marked reduction in exacerbations. However, response trajectories differed: patients with more evident T2 inflammation and without regular systemic steroid use improved earlier, whereas those with more severe, SCS‐dependent disease showed a slower but progressive response. Thus, while the magnitude of benefit may be preserved across phenotypes, the timing of improvement varies, with implications for treatment expectations, follow‐up, and interpretation of early response.

## Author Contributions

**Daria S. Fomina:** conceptualization, project administration, writing – original draft, methodology, investigation. **Sergey V. Fedosenko:** conceptualization, writing – original draft, methodology, writing – review and editing, investigation, formal analysis. **Аnton А. Chernov:** software, formal analysis, data curation, writing – review and editing. **Elena N. Bobrikova:** investigation, writing – review and editing. **Оlga А. Mukhina:** investigation, writing – review and editing. **Marina S. Lebedkina:** investigation, writing – review and editing. **Alexander V. Karaulov:** supervision, writing – review and editing, investigation. **Asel Yu. Nurtazina:** investigation, writing – review and editing. **Mariana A. Lysenko:** supervision, writing – review and editing, conceptualization. **Harald Renz:** writing – review and editing, supervision, validation.

## Funding

This study was funded by ANO “Moscow Center for Innovative Technologies”.

## Conflicts of Interest

D.S.F., S.V.F., A.V.K., O.A.M., and E.N.B. report personal fees from AstraZeneca, Novartis, Sanofi, GSK, Generium, and Teva. HR reports personal fees from Novartis, GSK, AstraZeneca, and Sterna‐Biologicals. The rest of the authors declare that they have no relevant conflicts of interest. All authors confirm that these disclosures do not affect the objectivity or integrity of the research presented in this manuscript.

## Supporting information


Supporting Information S1

## Data Availability

The data that support the findings of this study are available on request from the corresponding author. The data are not publicly available due to privacy or ethical restrictions.
