# Supplementary material for: Phenotypic Clusters of Severe Asthma and Response to Dupilumab in Real‐World Practice
Source: Clin Transl Allergy. 2026 Sep 15;16(9):e70198. doi: 10.1002/clt2.70198 (PMC13578103; doi:10.1002/clt2.70198)
Supplement: Supplementary file 1 [file CLT2-16-e70198-s001.docx]

**Supplementary Appendix**

**Table S1. Variable-Level Missingness of Baseline Features Included in Clustering Analysis**

| **Analyzed data (collected at baseline)** | **Patients with missing data** | |
| --- | --- | --- |
|  | **n** | **%** |
| Sex, male/female | 0 | 0 |
| Age, years | 0 | 0 |
| Smoking index (calculated for current and former smokers), pack/years | 0 | 0 |
| Conventional asthma phenotyping (allergic, non- allergic or mixed) | 0 | 0 |
| Age of asthma onset, years | 0 | 0 |
| Familial history of asthma, yes/no | 0 | 0 |
| History of severe atopic dermatitis, yes/no | 0 | 0 |
| History of “Atopic march”, yes/no | 0 | 0 |
| Sensitization to house dust mites, yes/no | 0 | 0 |
| Sensitization to pet allergens, yes/no | 0 | 0 |
| Sensitization to pollen, yes/no | 0 | 0 |
| Sensitization to allergens of non-pathogenic mold fungi, yes/no | 0 | 0 |
| Sensitization to food allergens, yes/no | 0 | 0 |
| Sensitization to ≥3 allergen groups, yes/no | 0 | 0 |
| ASIT history, yes/no | 0 | 0 |
| NSAID hypersensitivity, yes/no | 0 | 0 |
| Chronic rhinosinusitis with nasal polyps, yes/no | 0 | 0 |
| GERD, yes/no | 0 | 0 |
| Chronic urticaria, yes/no | 0 | 0 |
| Angioedema, yes/no | 0 | 0 |
| Cardiovascular comorbidity, yes/no | 0 | 0 |
| History of neoplasia, yes/no | 0 | 0 |
| Diabetes mellitus, yes/no | 0 | 0 |
| Kidney diseases, yes/no | 0 | 0 |
| Bronchiectasis, yes/no | 0 | 0 |
| Obesity, yes/no | 11 | 7.86 |
| ACQ-5 score | 4 | 2.86 |
| ACТ score | 1 | 0.71 |
| AQLQ score | 4 | 2.86 |
| Blood eosinophil counts | 2 | 1.53 |
| Total serum IgE level, kIU/L | 3 | 2.14 |
| Post-bronchodilator FEV1/FVC | 6 | 4.28 |
| Pre-bronchodilator FEV1, % predicted | 3 | 2.14 |
| FeNO value, ppb | 23 | 16.43 |
| Prior history of biological therapy, yes/no and details | 0 | 0 |

Notes: ASIT, Allergen Specific Immune Therapy; NSAID, non-steroidal anti-inflammatory drugs; GERD, Gastroesophageal Reflux Disease; FeNO, fractioned exhaled nitric oxide

**Table S2. Internal clustering validity metrics for candidate cluster solutions**

| **Candidate, k** | **Davies-Bouldin** | **Calinski-Harabasz** | **Silhouette** |
| --- | --- | --- | --- |
| 2 | 2.06 | 27.6 | 0.178 |
| **3** | **2.72** | **18.0** | **0.078** |
| 4 | 2.45 | 16.5 | 0.081 |
| 5 | 2.37 | 15.2 | 0.078 |
| 6 | 2.33 | 13.3 | 0.075 |
| 7 | 2.16 | 17.4 | 0.104 |
| 8 | 1.98 | 15.9 | 0.102 |
| 9 | 1.93 | 14.9 | 0.084 |

Notes: Lower Davies–Bouldin index and higher Calinski–Harabasz index and silhouette coefficient indicate better clustering performance. Metrics were evaluated for candidate solutions with k = 2–9. The 3-cluster solution was selected based on the overall balance of internal validation metrics and clinical interpretability, despite variability across individual indices. Clusters were derived using agglomerative hierarchical clustering on scaled baseline variables; t-SNE was used for visualization only and did not influence cluster assignment.
